# Immoral Intentions vs. Accidental Actions: Age Differences in Endorsements of Anger and Disgust

**DOI:** 10.1093/geroni/igab046.2841

**Published:** 2021-12-17

**Authors:** Alyssa Minton, Jason Snyder, Nathaniel Young, Verena Graupmann, Joseph Mikels

**Affiliations:** DePaul University, Chicago, Illinois, United States

## Abstract

Given that older adults value social harmony and selectively avoid negativity (Carstensen & Mikels, 2005), we investigated whether older and younger adults differentially react to scenarios in which someone intends to harm others compared to someone who accidentally harms others. Younger (n = 112, M = 26.23) and older (n = 113, M = 66.42) adults read 8 scenarios in which a perpetrator intentionally acts to harm someone else but is unsuccessful (Desire condition) or a perpetrator accidentally harms someone else (Consequence condition; Giner-Sorolla & Chapman, 2017). Endorsements of anger and disgust toward the perpetrators were measured on 7-point scales (1 = Not at all, 7 = Extremely). Emotion endorsements were submitted to 2 (age) x 2 (condition) ANOVAs. Anger (M = 4.81, SD = 1.58) and disgust (M = 4.82, SD = 1.54) endorsements were higher in the Desire relative to Consequence condition (M = 2.64, SD = 1.33; M = 2.49, SD = 1.29, respectively), F(2, 221) = 124.03, p < .001; F(2, 221) = 156.31, p < .001, respectively. Moreover, older (M = 5.17, SD = 1.61) relative to younger (M = 4.45, SD = 1.37) adults were disproportionately disgusted in the Desire condition, t(102) = 2.45, p = .016, but no age differences emerged in the Consequence condition. Results indicate that older (relative to younger) adults are disproportionately disgusted when judging a person who intends to harm others. Older adults may respond more strongly than younger adults to malicious perpetrators, as they intentionally upset social harmony.

